# Internal and External Load Variations in Young Students: Comparisons between Small-Sided Games and Small-Sided Games Combined with Strength Training during Physical Education Classes

**DOI:** 10.3390/ijerph18041926

**Published:** 2021-02-17

**Authors:** Juan Vicente Sierra-Ríos, Filipe Manuel Clemente, Israel Teoldo, Sixto González-Víllora

**Affiliations:** 1 Department of Physical Education, Arts Education, and Music, Faculty of Education, University of Castilla-La Mancha, 16001 Cuenca, Spain; juanvisr@gmail.com; 2 Escola Superior Desporto e Lazer, Instituto Politécnico de Viana do Castelo, Rua Escola Industrial e Comercial de Nun’Álvares, 4900-347 Viana do Castelo, Portugal; filipe.clemente5@gmail.com; 3 Instituto de Telecomunicações, Delegação da Covilhã, 1049-001 Lisboa, Portugal; 4 Centre of Research and Studies in Soccer, Universidade Federal de Viçosa, 36570-000 Viçosa, Brazil; israel.teoldo@ufv.br

**Keywords:** sports education, team sports, load monitoring, performance

## Abstract

The purpose of this study was to compare the effects of internal and external load in soccer small-sided games (SSGs) and a strength program based on CrossFit combined with SSGs for 7 weeks. Fifty-five students participated in this research (age: 9.04 ± 0.19 years) and were randomly assigned to SSGs (*n* = 27) or strength combined with SSGs (*n* = 29) group. Two sessions/week were implemented. The results revealed that internal load on SSGs promoted higher levels (*p* ≤ 0.001; d = 0.35) of light physical activity (PA) (12.24 cpm) compared with strength combined with SSGs (11.46) and % heart rate (%HR) max (*p* = 0.002; d = 0.48) between SSGs (96.21) regarding strength combined with SSGs (92.09). On external load, significant differences appear in total distance (*p* ≤ 0.001; d = 0.80) on SSGs (1326 m) compared with strength combined with SSGs (1004 m) and mean velocity (*p* ≤ 0.001; d = 0.63) in both groups; 2.71 km/h on SSGs and 2.26 km/h on strength combined with SSGs. The SSGs seem to be more appropriate at the beginning of sessions, but as the weeks advance, strength combined with SSGs results in improved internal load compared with SSGs.

## 1. Introduction

Physical education at present is oriented towards the improvement of health, in which it is necessary to carry out a series of approaches and trends. The scientific literature agrees with the needs of children to be physically active in physical education classes [1]. Schools must contribute to achieving the goal of sixty daily minutes of moderate and vigorous physical activity as recommended by the national health organization [2]. For this reason, an initiative has arisen that suggests that children participate in a minimum number of physical education classes per week [2,3]. It is recommended that elementary schools provide 225 min of physical education per week. To promote physical sporting activity in educational centres, it is necessary to modernize the physical education curriculum and, secondly, the teaching style. The purpose of physical education should be to teach students to take responsibility for the care of their health-related physical condition [4].

As physical education classes are commonly sports-oriented (i.e., focused on specific sports); most of the time, the students are enrolled in drill-based tasks. Among those drill-based tasks, small-sided games (SSGs) are one of the most popular drills used by physical education teachers to promote both physiological/physical and tactical/technical stimulus [5]. These games are typically conditioned by different task constraints that act concurrently to explain the acute response of players [6]. Among others, the most common task conditions by teachers are as follows [6]: (i) format of play (i.e., number of players involved and numerical relationships); (ii) pitch configuration (e.g., width per length ratio, individual area per player, pitch geometry); (iii) scoring method (e.g., regular vs. smaller goals; other types of scoring); (iv) movements restrictions (e.g., the ball touches limitations; actions limitations); (v) specific instructions (e.g., type of marking; type attack); and (vi) regimen of training (e.g., type and modality of exercise, work-to-rest ratio).

Typically, SSGs (namely between 1 vs. 1 and 4 vs. 4) are very highly demanding, as the acute responses of heart rate are usually above 85% of heart rate maximum [7,8]. Considering the highly demanding impact of these games, they are commonly used in an intermittent modality in which repetitions may vary between 1 and 5 min, with work-to-rest ratios between 1:1 and 1:2 [9,10]. As these games normally tax the cardiorespiratory system, they are highly effective in improving aerobic performance in young players [11,12,13]. In fact, in a systematic review with meta-analysis, it was found that SSGs were similarly effective to running-based endurance methods in improving aerobic performance [14].

Despite the apparent effectiveness of SSGs for improving aerobic performance, it seems that is not enough to reach other physical qualities that are also determinant for health and fitness of youth. The effectiveness in improving neuromuscular-based qualities (e.g., strength, power) is low [15]. Additionally, the great intra- and inter-variability of SSGs stimulus, as well as the low demanding in high intensity running demands (e.g., high-speed running, sprinting), are threats that must be considered in physical education classes by teachers [16]. For this reason, specific strategies for developing the neuromuscular status of youth students should be considered.

The World Health Organization and the United States Department of Health and Human Services support the promotion of muscle-strengthening activities in addition to aerobic activity as part of their physical activity guidelines for children and adolescents [17,18]. Any training program that combines high-intensity resistance exercise with high cardiorespiratory demands could be a training method of high interest for individuals with the desire to improve their fitness [19]. It is thus the case that strength training emerged as an appealing time-saving alternative when compared with traditional training programs. It has been shown that HR elicited increases in aerobic capacity and anaerobic performance [20,21].

Comparing the effect of SSGs and the combined strength training program and SSGs interventions in different physical education sessions will also help researchers to better understand the effects of manipulating the order of these training programs.

Because of the limited research related to both training programs [16,22,23] in young soccer players, as well as the fact that no related studies have been found in the school and educational setting, the objective of this research is to analyze the training load (internal and external) in schoolchildren divided into two groups during physical education lessons. We emphasize the importance of the internal load, which is more interesting to plan both training programs. The first is based on SSG and the second on strength training combined with SSG. Through this research, it is possible to understand the magnitude of the effects of reduced games as well as their combination with strength training to identify the main variables that can be benefited by models related to the game. To do this, we will analyze the effects on PA levels during physical education sessions. Finally, the knowledge of the real effects will help to identify the most appropriate strategies on the internal and external load to promote higher levels of physical activity, heart rate, distance travelled, and speed.

## 2. Materials and Methods

### 2.1. Study Design

This study followed a parallel study design consisting of physical education sessions on a soccer didactic unit with based on SSGs with teaching games for understanding approach (TGfU) and one with strength training of CrossFit and soccer SSGs.

### 2.2. Setting

The study was realized in an elementary primary school. Both experimental groups had two intervention sessions a week for a period of seven weeks. The experimental approach occurred from January to March with two groups of the fourth grade. As a result, fourteen experimental sessions were employed with a mean duration of 45 min each. Physical activity (PA) levels were monitored by accelerometers and heart rate (HR) by Polar Team Pro^®^.

A physical education teacher implemented both teaching approaches (SSGs and strength combined with SSGs). The players who participated in the strength training program combined with SSGs group developed the activities using the analytical technique used so far, following the structure of (a) warm-up with continuous running and joint mobility and (b) strength part based on TABATA CrossFit training. This type of CrossFit training is based on eight different strength exercises using your body weight as load, for four minutes divided in 20 s of working with 10 s to rest. Pupils did two series with two minutes of rest between them. After strength training, pupils did 20 min of soccer training based on SSGs with technical and tactical exercises according to the objective; and (c) to conclude with stretching exercises. Table 1 shows the eight different strength exercises based on CrossFit training.

The players of the group based on SSGs developed the sessions using the following structure: (a) warm-up with a playful game with the ball; (b) an initial reflection was introduced in which the objective of the session was exposed; (c) the modified games were carried out practically and experientially, and the learning that arises was favoured. The exercises based on small-sided games were formatted as 3 versus 3, a game zone of 200 m^2^ (10 m × 20 m) with a goal on each side of the field of two meters, as well as no limit of contact with the ball.

Finally, the training concluded with a final reflection in which the aspects learned were transferred.

### 2.3. Participants

The present study used a non-probabilistic inter-subject case design for the convenience of two classes. Fifty-five pupils (age 9.04 ± 0.19) of physical education were assigned to two intervention groups; one group (*n* = 27; 19 boys and 8 girls) participated on soccer didactic unit in SSGs based on TGfU, and the other group (*n* = 29; 17 boys and 12 girls) in strength training of CrossFit and soccer SSGs.

Table 2 shows information on the age, height, sitting height, weight, and body mass index of each team. The current study described 14 training sessions split by 7 weeks. The players had the same competitive and playing skill level. Players were randomly assigned to the groups and all the participants were present in all training sessions using accelerometer and Polar Team Pro^®^.

### 2.4. Variables

#### 2.4.1. Independent Variables

The study was established in two groups within the same school year (fourth grade of primary school) and divided according to the class to which they correspond. Randomization has not to be performed as no significant differences were found comparing baseline variables.

#### 2.4.2. Dependent Variables: Internal Load

The internal load was analysed by physical activity levels and heart rate frequency. Physical activity levels were measured on counts per minute in variables of light, moderate, vigorous, and moderate to vigorous physical activity (MVPA). Heart rate was analysed as beats per minute as a total percentage in variables minimum, mean, and maximum. To measure this variable, wGT3X accelerometers were implemented for PA levels and Polar Team Pro^®^ for HR.

#### 2.4.3. Dependent Variables: External Load

This variable was analysed by total distance and mean velocity. Both variables were quantified by Polar Team Pro^®^. The total distance variable was used as a unit of measurement in meters and the mean velocity in kilometres per hour.

### 2.5. Data Sources

Anthropometry measurement was made according to Kushner protocol [24]. The body mass and height were measured twice with a five-minute interval between measurements. The body mass was measured at the nearest 100 g using a calibrated digital scale (SECA Model 861, Vogel and Halke, Hamburg, Germany) with slightly dressed children without shoes. The height was measured to the nearest millimetre using a wall-mounted stadiometer, with children standing directly against the wall without shoes, to align the spine with the stadiometer. The head was placed so that the chin is parallel to the floor. The mean of the two measures of body mass and height was used to calculate the BMI as the body mass in kilograms divided by the square of the height in meters (kg/m^2^).

The wGT3X accelerometers (three axes, 100 Hz, Actigraph) were used to measure the PA levels during training sessions. The instrument was previously tested for its validity and reliability to measure PA levels [25]. The device was placed on the right hip, above the iliac crest, near the centre of gravity [26]. The accelerometers were programmed in 100 Hz and exported in the epoch of 1 s. The cut points obtained from the accelerometers [27] were sedentary PA 0–100 counts per minute (CPM), light PA 100–2295 CPM, moderate PA 2296–4012 CPM, and vigorous PA ≥ 4013 CPM. The data were analysed using the Actilife 6.0 software (Pensacola, FL, USA).

Polar Team Pro^®^ recording and monitoring system. To record the HR and the distance travelled during the implementation of the tasks in an objective way. The Polar Team Pro^®^ system is composed of the following: (I) a sensor that allows GPS recording (10 Hz), an accelerometer, gyroscope, and digital compass (200 Hz); (II) a chest band weighing 60 g; (III) a sensor synchronization and charging station (Polar Team Pro^®^); and (IV) an application compatible with an iPad^®^ to empty the information in the Polar^®^ cloud (Polar Team Pro App^®^). Once the profile of each participant had been configured with the information related to age, weight, and height, a personal sensor number was assigned to each one to specifically record the cited variables, considering the anthropometric characteristics of the student.

The Polar Team Pro^®^ telemetry system (Polar Team System, Polar Electro Oy, Espoo, Finland), which includes GPS operating at a sampling frequency of 1 Hz and incorporates a triaxial accelerometer at 1 Hz, was used to record the total distance travelled (Dtot) (m × min ^−1^), distance to each effort intensity (3–5 categories of effort intensity) (m × min ^−1^), and maximum speed reached (Vmax) (m × s ^−1^). The system is accurate and reliable as revealed in recent independent research [28].

### 2.6. Study Size

Sample size calculation was made for an alpha of 0.05 and power of 0.8. The results indicated a recommended sample of 49 or more measurements/surveys is needed to have a confidence level of 95% that the real value is within ±5% of the measured/surveyed value.

### 2.7. Quantitative Variables

Pupils from the same school and grade participated in the study (one group underwent small-sided games based on the TGfU, and the other group underwent training on strength and small-sided games). The inclusion criteria were as follows: (i) the players had previous experience in physical education soccer classes, but not with the TGFU. The players regularly performed two weekly training sessions (~45 min each session).

To ensure high quality, the research has followed every ethical standard throughout including data collection, data analysis, and the publication process (including the confidentiality of the personal information in the data). Besides, players to be included in the research must provide informed consent under the Declaration of Helsinki, indicating that their voluntary participation was obtained from and their legal representatives after the explanation of the experimental protocol and the possible benefits and risks of the research project. The authors/researchers have paid extraordinary attention to those requirements related to privacy and confidentiality and informed consent. Finally, these requirements were adapted to the Social Sciences and educational context.

### 2.8. Statistical Analysis

The data were analysed with the statistical program SPSS version 24.0 (Chicago, IL, USA). To check the normality of the variables, the Shapiro–Wilk test was used, because the sample was less than 50. As the variables presented a normal distribution (*p* > 0.05) and homogeneity in the Levene test (*p* > 0.05), parametric tests were performed. The PA levels, heart rate, total distance, and mean velocity were adjusted by body mass index (BMI) as a covariate to control for possible effects on physical activity levels. All variables were compared using multivariate analysis (MANCOVA). Additionally, Cohen’s d was used to calculate effect sizes [29]. The magnitude of changes was classified based on the following thresholds: trivial (d < 0.2), small (0.2 ≤ d < 0.5), medium (0.5 ≤ d < 0.8), or large (d ≥ 0.8). Bonferroni was used to control the type I error. The level of statistical significance was set at *p* ≤ 0.05, with a confidence interval of 95%.

## 3. Results

The MANCOVA results examine each training load type of PA, heart rate, total distance, and velocity between both training programs.

Table 3 shows the mean results of the internal load (PA level and HR level) and external load (total distance and mean velocity) in each of the groups (SSG and strength combined with SSG) monitored over the sessions. The pupils’ group realized that SSG had significant differences on light PA, total distance, and mean velocity compared with the strength combined with SSGs group.

Starting with internal load, only PA levels in light PA show significant differences in small-sided games of 12.24 compared with strength training program plus SSGs. Moderate PA and MVPA in the strength training program are higher than in SSGs, but not significantly. Finally, heart rate is better in the variables minimum and maximum in the SSGs group compared with the strength training combined with SSGs program, but not significantly. Heart rate mean is higher in the strength training combined with SSGs program than in the SSGs program.

Nevertheless, analysing external loads of SSGs presented significant differences in total distance and mean velocity compared with the strength training combined with SSGs program.

**Figure 1:**
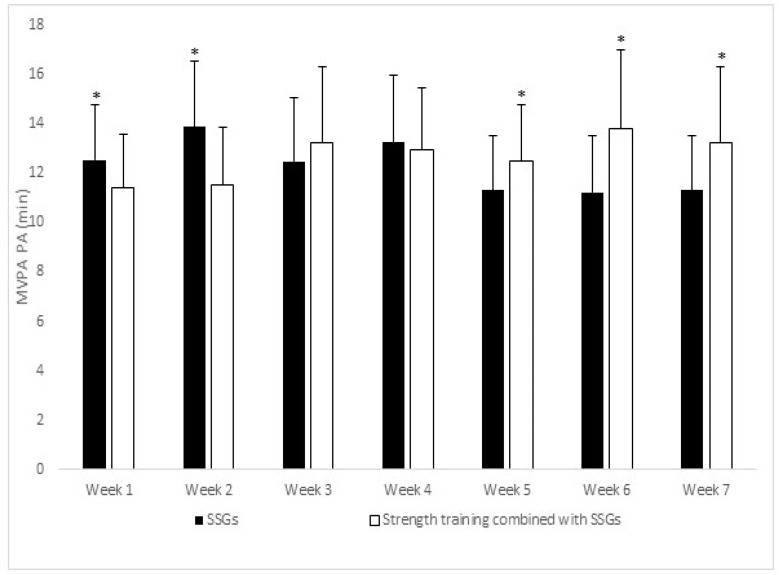
Moderate to vigorous physical activity (MVPA) by weeks. * denotes *p* < 0.05 differences. SSGs, small-sided games.

Analysing the moderate to vigorous PA, Figure 1 shows significant differences between small-sided games and strength training combined with SSGs on weeks one and two, but in weeks five, six, and seven, significant differences appear in the strength training combined with SSGs program compared with the training program based on SSGs only.

**Figure 2:**
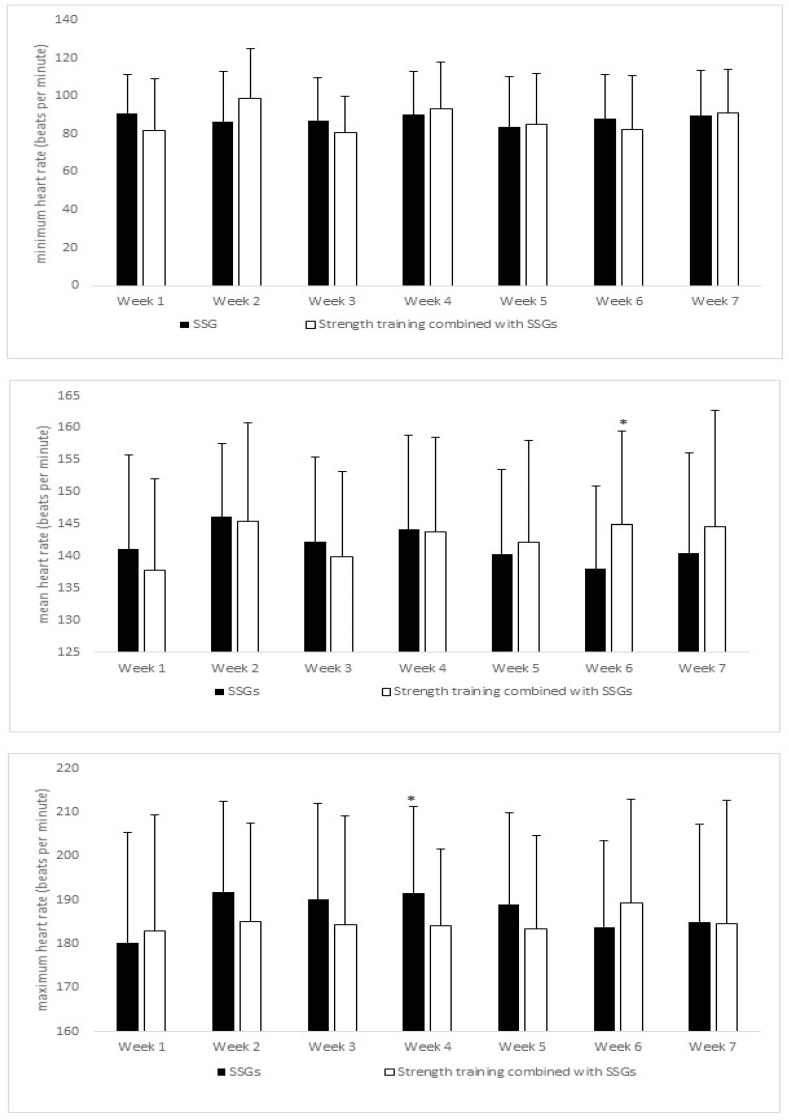
Heart rate activity by weeks (minimum, medium, and maximum). * denotes *p* < 0.05 differences.

Regarding heart rate, Figure 2 illustrates the minimum heart rate between two training programs. In this case, there are no significant differences. For the mean heart rate for seven-week implementation programs, only in week 6 does the strength training program combined with SSGs show significant differences compared with SSGs only. Finally, the last variable analysed on internal load was the maximum heart rate, which showed significant differences in week 4 in SSGs compared with the strength training program combined with SSGs.

**Figure 3:**
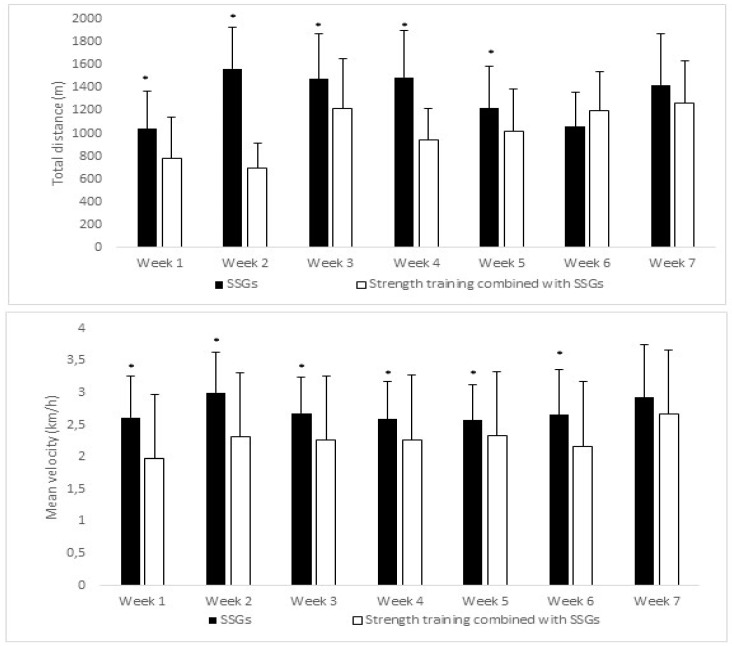
Total distance (m) and mean velocity (hm/k). * denotes *p* < 0.05 differences.

On the other hand, the external load variables were analysed. Figure 3 shows significant differences between SSGs and the strength training program combined with SSGs in weeks one to five. The variable of velocity by weeks shows significant differences in SSGs compared with the strength training program combined with SSGs in weeks one to six.

## 4. Discussion

The main objective of the study was to compare the training load (internal and external) in the school environment using accelerometry and Polar Team Pro^®^ GPS through the implementation of two soccer training programs (SSGs and strength training combined with SSGs). According to the initial hypotheses, SSGs showed significantly higher levels of internal load concerning the variables of light physical activity and the percentage of maximum heart rate. It can also be verified that, as the intervention progresses, the levels of physical activity are significant in the group that performs the strength program combined with SSGs. Regarding the external load, SSGs showed significant differences in the total distance and the mean speed in the strength training program combined with SSGs. However, no significant differences were found in the internal load variables between both training programs in physical activity levels (moderate, vigorous, and MVPA) as well as heart rate (minimum, mean, and maximum beats per minute).

### 4.1. Internal Load

Regarding the internal load, the present research obtained a mean HR in both programs of 143 bpm and a %HR higher in SSGs compared with strength training combined with SSGs. This difference could be because the group of SSGs performed a greater number of movements and accelerations, producing an increase in heart rate compared with the group of strength training combined with SSGs. Compared with previous research in the variables of HR and a%HR, the work of [16] obtained a HR between 173 and 176 and a %HR max of around 88% in SSGs in 3 × 3 and 4 × 4 formats in young soccer players. Owing to the smaller size of the practice space (our study’s SSGs were approximately 20 × 10 m in size), our study obtained better results in both physical training programs, with 191 bpm and 96% HR max, and in SSGs, with 189 bpm and 92%, respectively.

Analysing the internal load variables for the age of the players, the age of our participants can condition the response of the internal load of the players as well as the training time, because, as the duration gets shorter, the intensity of the training increases, causing an increase in HR. Castellano and colleagues (2016) [30] found that, after 75 min of training, children at similar ages (12 U and 13 U) showed an inter-individual variation in HRmax values between 80 and 90%, lower than in our study. Other results with young soccer players are found in Rojas-Inda [31], who found a mean HR of 175 bpm and a max HR of 193 bpm, using 4 × 4 SSGs formats, which is higher than our study for mean HR, but similar for HR max.

In physical education sessions based on SSGs, improvement in physical condition concerning internal load could be demonstrated compared with other training programs. For interval running [32], heart rate was recorded with a percentage of HRmax of 89.6 SSG, interval running with HRmax above 80%, and aerobic training above 90% of HRmax. These results are similar to those in our study and could demonstrate the importance of using SSGs to improve internal load fitness compared with other training programs such as interval running.

In a context like ours, short sessions with SSGs, as well as interval running, can be performed with high average intensity and with periods of near-maximum aerobic load. The integration of all training contents within a given microcycle is considered with overall performance in mind [12]. This additional physiological stress should be considered taking note of the other physical and technical/tactical sessions to avoid overload and optimize adaptation [32].

In summary, SSGs improve the internal load on HR and HR max in young students, creating positive fitness effects. Finally, we highlighted that, when the SSGs are performed in a short time with high intensity, the students move constantly, producing a heart rate and levels of physical activity from moderate to vigorous high.

### 4.2. External Load

The main conclusion was the significant differences in external load between both programs (SSGs and resistance training combined with SSGs). The reason for the difference between both training programs may be that, for the group that practised the strength program, the CrossFit exercises were performed without displacement, with fewer exercises such as jumps or push-ups during that period, influencing the distance travelled and the speed of travel. Future studies could analyse external loading with CrossFit exercises performed with displacement.

Comparing the external load with previous studies, Köklü and colleagues (2020) [16] obtained results for the total distance between 1900 and 2300 m. These results are higher than those of our study, that is, 1000 and 1300 m. In our study, the activity time was 45 min, while for Köklü and colleagues (2020) [16], the activity duration is 90 min. Furthermore, the size of the SSGs format of Köklü and colleagues (2020) [16] was larger (20 × 30 m), causing an increase in total distance. With SSGs formats like the one in our research, Castellano and colleagues (2016) [30] obtained a total distance lower than in our study (530 m). Besides, we obtained results lower than those of Sierra-Díaz (2020) [33] with similar players (4 vs. 4) and an SSGs size greater than that in our study obtained an average of 658 m. Finally, in SSGs formats with a greater number of players (7 vs. 7) and relative area per player (100 m^2^) such as in Rojas-Inda (2018) [31], a total distance between 1700 and 2300 m was achieved, greater than in our study.

### 4.3. Study Limitations, Future Research, and Practical Applications

This study has some limitations; firstly, the sample is small, with only fifty-five players participating. Moreover, the physical education sessions need adaptation to consolidate the SSGs with TGfU and the CrossFit exercise combined with SSGs. Future studies should be expanded in the extracurricular field, with a larger sample with annual planning. Moreover, a comparison between the categories of initiation stages (U-8 and U-10) comparing physical and physiological variables with the tactical–technical performance of the game, as well as with psychosocial aspects, such as the degree of motivation that underlies the participation of the SSGs in physical education classes [34], would be interesting, as would an analysis of the difference between genders [35], so the subject of physical education classes is a mix. Besides, it is important to analyse game performance or mental fatigue, causing a compensatory increase in physical performance [36]. Finally, the use of tools to quantify the load such as inertial devices such as WIMU^TM^ or the measurement of subjective load through the Integral System for the Analysis of Training Tasks (SIATE) would be very useful for its registration in the absence of technological material [37]. As practical implications, the results of this study allow us to highlight the importance of SSGs, which encourages a reduction in sedentary activity.

## 5. Conclusions

The research allowed to understand the effects of training programs based on SSGs and the strength training program combined with SSGs on internal and external load in a soccer didactic unit in physical education classes. The main results were the significant differences in the group of SSGs in the internal load to light PA, % HRmax, and external load, of both the total distance and the average speed, compared with the strength training program combined with SSGs. As the training sessions progressed, when the workload accumulated, the strength training program combined with SSGs improved physical activity levels and heart rate relative to practising only SSGs.

It is relevant to educate people in physical education classes and in training programs through combining strength and SSGs in order to remind the player of the importance of improving their physical activity and reducing sedentary lifestyle.

## Figures and Tables

**Table 1:** Strength exercises based on CrossFit training.

1. Burpees
2. Skipping
3. Horizontal plate
4. Jump squat
5. Push-ups with knee support
6. Box Jump
7. Triceps curls
8. Side plank

**Table 2:** Information of subjects about age, height, sitting height, weight, and body mass index (BMI) according to groups (small-sided games (SSGs) or strength combined with small-sided games).

	SSGs (*n* = 27)	Strength Training Combined with SSGs (*n* = 29)
	(M ± SD)	(M ± SD)
Age (years)	9.04 ± 0.19	9.11 ± 0.31
Height (m)	1.37 ± 0.08	1.37 ± 0.06
Sitting height (cm)	69.08 ± 3.89	69.13 ± 3.86
Weight (kg)	37.40 ± 10.93	36.48 ± 7.72
Body Mass Index (BMI) (kg/m^2^)	19.54 ± 4.17	19.20 ± 3.22

Note. n: sample; M: average; SD: standard deviation; m: meter; cm: centimeter; kg: kilograms.

**Table 3:** Internal load (PA and HR) and external load (total distance and velocity).

		SSGs (*n* = 27) (M ± SD)	Strength Training Combined with SSGs (*n* = 29) (M ± SD)	Mean Differences	*p*	d
Internal load	Light PA (cpm)	12.24 ± 2.19	11.46 ± 2.23	17.61	<0.001	0.35
	Moderate PA (cpm)	4.22 ± 0.99	4.39 ± 1.11	3.48	0.062	0.16
	Vigorous PA (cpm)	8.12 ± 2.21	8.00 ± 2.05	0.47	0.492	0.05
	MVPA PA (cpm)	12.35 ± 2.58	12.63 ± 2.80	1.66	0.198	0.10
	HR min (bpm)	89.25 ± 22.49	87.94 ± 26.05	0.406	0.524	0.05
	HR mean (bpm)	142.30 ± 12.11	143.87 ± 14.17	1.98	0.159	0.09
	HR mean (%)	71.18 ± 6.14	70.35 ± 5.61	1.13	0.289	0.14
	HR max (bpm)	191.69 ± 15.28	189.07 ± 18.66	3.31	0.069	0.15
	HR max (%)	96.21 ± 8.03	92.09 ± 8.77	9.61	0.002	0.48
External load	Total distance (m)	1326.97 ± 402.65	1004.65 ± 396.81	96.17	<0.001	0.80
	Mean velocity (km/h)	2.71 ± 0.63	2.26 ± 0.77	57.44	<0.001	0.63

Note. n: sample; M: average; SD: standard deviation; PA: physical activity; MVPA: moderate to vigorous physical activity; cpm: counts per minute; HR: heart rate; bpm: beats per minute; %: percentage; km/h: kilometres per hour; m: meters; d: Cohen’s d.

## Data Availability

The data presented in this study are available on request from the corresponding author.
